# System dynamic modelling of electricity planning and climate change in West Africa

**DOI:** 10.12688/aasopenres.12852.2

**Published:** 2018-10-04

**Authors:** Abiodun S. Momodu, Lucy Kivuti-Bitok

**Affiliations:** 1 Centre for Energy Research and Development, Obafemi Awolowo University, Ile-Ife, 220005, Nigeria; 2 School of Nursing Sciences, College of Health Sciences, , University of Nairobi, Nairobi , Kenya

**Keywords:** System Dynamic Modelling, Climate Change, Energy, Policy, West Africa

## Abstract

**Background**: It is imperative to develop an efficient strategic approach to managing the push-pull factor in economic development, particularly as relates to climate change and energy interactions in the West African Region. This article demonstrates the use of System Dynamics Modelling (SDM) for that purpose; to manage the development of energy growth with reduced impact in regards to climate change. The complexities of energy planning in relation to climate change necessitates the need for the tool to examine low carbon economy mixed with traditional approaches of planning.

**Methods**: Vensim DSS version 6.2 was used to develop the model. WAPP member country level data elicited from WAPP and ECOWAS Regional Electricity Regulatory Authority (ERERA) serves as the set of basic data used to develop and run the main model. These were complemented with other data elicited from various journal articles and internet sources. These include population and its average growth rate, GDP, per capita income, average per capita electricity demand, electricity generated, average electricity tariff, generation technology type, amongst others.

**Results**: SDM demonstrates the capability to understand the theoretical frame for trade-offs between economic development and climate change, by handling the nonlinear relationship between generation adequacy and greenhouse gas (GHG) emission reduction for better targeted strategic regional intervention on climate change.

**Conclusion**: The primary goal of this paper was to demonstrate the use of SDM to aid in resource planning in an inexpensive way to examine low carbon pathway. With the SDM, the goal of low carbon pathway in the energy system was achieved without the cost of controlled trials.

## Introduction

Energy, particularly in electrical form, is one of the most important value-adding commodities to sustainable development. Its planning is therefore very important, if the characteristic of adding value to economic development is to be harnessed. As an example of its importance, in recent years, electricity comes as a panacea to the use of petroleum products in the transportation sector to reduce greenhouse (GHG) gas emissions. Electric vehicles are now technically feasible and economically viable, and various governments have announced the specific dates to eliminate the use of petroleum based vehicles (see report from CNN). This development serves to accentuate the need for electricity planning to increase accessibility as priority (see Research and Innovation Roadmap from ENTSOE). Although accompanied with several challenges, such as 1) complexities in generation and wheeling capacity; 2) long periods and delays in construction; 3) difficulty in storing large amounts of electricity; 4) irreversibility of project investment, thus posing severe setbacks to development. Planning in the electricity sector is crucial for development. These factors, coupled with the aging of generating plants in the electricity sector in West Africa, creates high levels of uncertainty, therefore, making it difficult for the energy system in the region to achieve maximum operational efficiency ^1^. These challenges notwithstanding, it is expedient to understand the dynamics involved in the West African electricity sector for policy and proper planning. Application of System Dynamics (SD) has been extensively used both as a method and tool to aid in resource planning in a number of sectors including the electric power industry ^2– 5^.

On the other hand, climate change is multi-faceted, and constitutes a grave danger to the world (see United Nations Low carbon development strategies). Climate change is driven mainly by CO _2_ emissions. Conversely, economic growth demands energy consumption. In turn, level of energy consumption and the makeup of the energy basket drive CO _2_ emissions. To strike a balance and reduce emission, there is need to lower energy consumption. Lower energy consumption can come about from technological progression, lower economic growth, or demographic changes, or by moving the composition of the energy basket to sources with lower emission content. These factors have a non-linear relationship, requiring an in-depth understanding of designing effective climate policies.

Many approaches have been adopted to examine these complex interactions. These include comparative studies that employ decomposition techniques to analyze the drivers of CO _2_ emissions, covering only the most recent decades ^6– 10^. According to these studies, the greatest driver of CO _2_ emissions is economic growth. There is however disagreement on the relative importance of other factors depending on the period of analysis, the applied methodology and the level of regional aggregation. Country differences in population size affluence and technology were further masked by aggregated global or regional analyses. Also, the short-time span of these studies made them unable to fully capture how drivers change in importance over time, hence this study.

The objective of this study is to present System Dynamic Modelling (SDM) approach that caters for the demand of making efficient strategic policies to manage the push-pull factor in climate change and energy use as it concerns the drive for economic development. SDM addresses the challenges of complexities and non-linearity in the push-pull forces of cause and effects in the use of energy for economic development and the attendant CO _2_ emissions over time. Further, SDM addresses issues of periodicity and aggregation of data, taking into consideration both hard and soft variables ^11^. The modelling approach examines the relationship amongst various factors in the complex system of energy-climate change interactions as concerns economic development. SDM is an extensively used method and a resource planning tool for electricity industry and other sectors, with capability to clearly assess the dynamic structure and behavior of systems ^5^.

The field of SD introduced by Jay Forrester in the 1960s ^12^ emerged from engineering feedback control systems and electronics ^8^. Since then, SD has been relevant in modelling field since 1950s. SDM is a well-established approach to visualizing and analyzing complex systems, including dynamic feedback systems with interactions between several influencing factors and elements within a system. It is used to x-ray systems in other to know what is within the system that creates a cause and effect relationship over time ^10^. Thus, this justifies the reason for adopting SD in this study.

## System Dynamics Modelling

**Figure 1.**
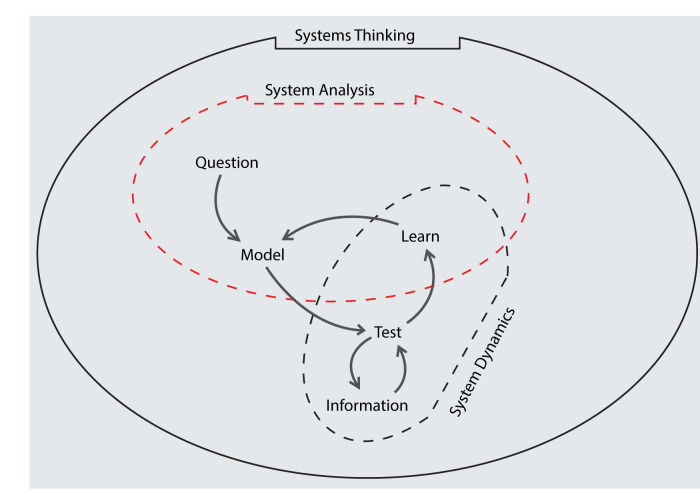
Explaining the concept of System Dynamics within System Thinking ^13^.

The basis for SDM is Systems Thinking. The world is a complex system. System thinking gives room to view it with the understanding that "one thing alone is not enough" and that “everything is connected to everything else.” This represents the mindset and philosophy of thinking about whole systems rather than symptoms and event sequences. Systems Thinking has an “eagle’s view”, identifying causalities that gave rise to events and histories. In this process, system boundaries are defined and communicated. Following this is system analysis: taking apart the system in order to understand its causalities, detect and discover what structural arrangement is in them. This yields an understanding of the effects emerging from the flows and accumulations due to the causalities acting in the system. These descriptions are what generates System Dynamics (SD) as shown in Figure 1. The use of SD involves assessing the performance of reproducing the events and histories of the system in order to foresee and forecast its future behavior. In this context, it is important to describe some important concepts in SD, which include delays, feedback, causal loop diagram (CLD) and stock and flow diagram (SFD).

Dynamics are created from delays, as these give systems inertia, generate oscillations ^13^. These often are responsible for trade-offs between the short- and long-run effects on policies ^14^.

Feedback could occur in any of two ways: either as positive (reinforcing) feedback loops or negative (balancing) loop. Self-reinforcing loops are called positive (reinforcing) feedback. They seek to grow exponentially forever, and since no quantity can grow forever, there must be limits to growth. The self-correcting loops or negative (balancing) feedback are what limit growth as they counteract change ^13– 15^. Further description of how loops work in SDM is given in Bitok-Kivuti, Momodu and Pokhariyal ^16^.

An important tool for projecting SD is CLD. It represents causalities and feedback structures of complex systems. CLD quickly captures hypotheses about the causes of dynamics; elicit and capture the mental models of individual teams and communicate the important feedbacks believed to be responsible for a problem ^14^. The other important tool in SD is stocks and flows diagram (SFD). Modelling in SD is predicated on stocks and flows, along with feedback as the central concepts of dynamic system theory ^14^. Stocks are accumulations of anything that can be counted, give systems inertia and provide them with memory. Delays are created in stocks through accumulating the difference between inflow and outflow ^14, 15^.

**Figure 2.**
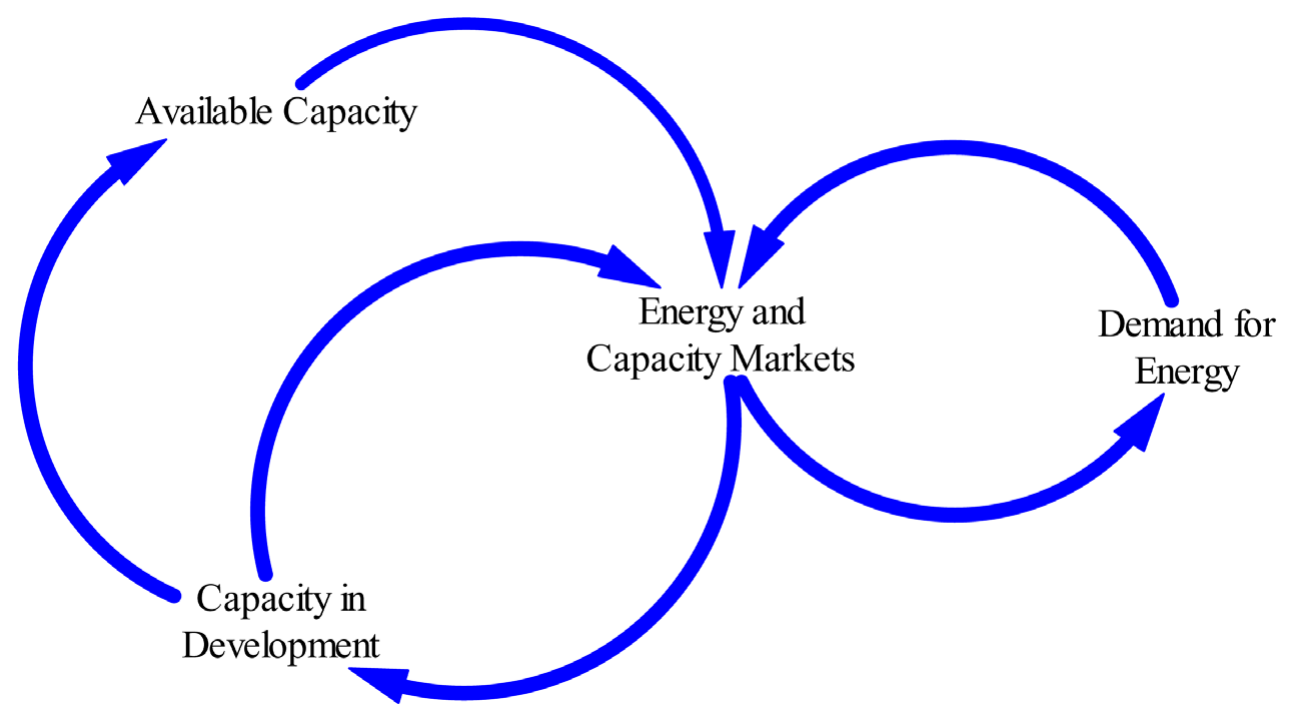
An Example of a CLD in Energy and Capacity Markets (adopted from Olsina ^17^).

**Figure 3.**
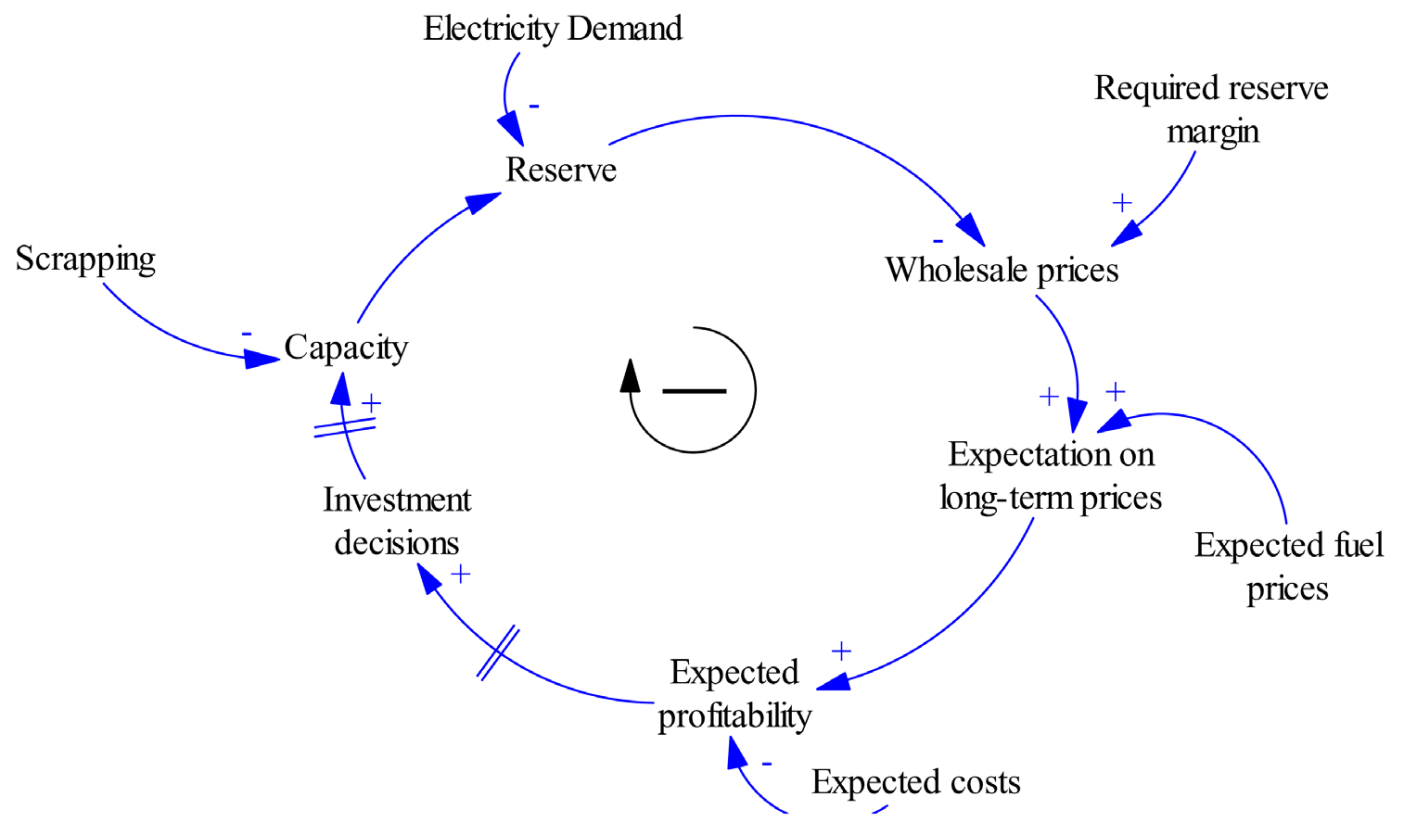
Causal-Loop Diagram of a Typical Electric Power System (adopted from Olsina ^17^).

Basic feedback structure depicting a simplified causal-loop for electric power system as adopted from Olsina ^17^ is presented in Figure 2 and Figure 3 respectively. These provide an overview of the system’s dynamical structure and to guide further discussions when modelling the different system components. The diagrams show the basic balancing feedback that governs the long-term development of any power market. The existing capacity plus the additions of new capacity, the scrapping/decommissioning of old power plants and the current system demand will determine the new reserve margin. With this therefore, the system becomes self-balancing and resembles the negative feedback loops commonly encountered in control systems. This balancing mechanism is responsible for maintaining an adequate reserve margin to ensure a reliable electricity supply.

However, this causal-loop-diagram (CLD) is useful to represent the causal relationships and the system balancing feedbacks responsible for adjusting the production capacity, it is not capable to show explicitly stock-and-flow structures embedded in the system. The stocks-and-flow-diagram (SFD) shows important variables controlling rates of flow into stocks, making the issue of capacity adjustment mechanisms clearer.

## SDM of climate change - energy interactions in West Africa

Energy is an essential element needed for the rapid transformation of West Africa to pull it out of its current under-developed state. However, to avoid the pre-industrialization trajectory ^1^, there is need to understand what trade-offs exists between economic growth and development aspiration as well as that of climate change issues. Though large-scale economic development is needed to pull millions of citizens out of abject poverty, a "business-as-usual" approach would exacerbate the problem of climate change with potentially irreversible long-term consequences. Low carbon development strategies (LCDSs) have attracted the interest in the climate negotiations as a soft alternative to voluntary or obligatory GHG emission reduction targets in developing countries ^18^.

West Africa is made up of 15 countries, with 14 of these in the West African Power Pool (WAPP). The WAPP system is made up of only four types electricity generation technology in its stock, categorized based on fuel. The technologies within these fuel types also vary in terms efficiency, emission and capacity factor. In 2015, this was made up of oil about 14%, coal, 0.3%, natural gas 48.5%, with hydro rounding it off at 37.2% ^2^ of production. Nuclear technology is absent in the mix. Population size, economic activity, lifestyle, energy use, land-use patterns, technology and climate policy are notable anthropogenic greenhouse gas (GHG) emissions drivers ^19^; meaning new approaches, such as low carbon pathway being examined in this article, are needed to control future emissions ^19^.

A number of studies have been conducted examining electricity and climate change in West Africa. A study by Gnansounou *et al.* ^20^ examined strategies on electricity supply and climate change, reporting on the evolution of regional electricity market on the basis of two strategies - “autarkical” and “integration ^2^”. It recommends integration strategy that allows for fast withdrawal of the aged power plants and “ *the integration of new investment projects to bring about additional benefits in terms of reduced capital expenditures, lower electricity supply cost and the enhanced system's reliability compared to the autarkical strategy*”. It did not examine the climate change and cost impacts. Another study on WAPP ^21^ developed “ *models to understand the long-term interactions between investment and performance in the electric power system*”. It shows that WAPP interconnection has a “ *clear impact on the local system prices and investments in new construction but there will still be large regional variations in prices and new construction*”. A third study ^22^ assesses “ *extreme temperatures and heat waves impacts on electricity consumption in some cities in West-Africa*”. It reports that “ *electricity consumption trends in the cities examined match extreme temperatures evolution well*”. The SD study by Momodu *et al.* ^2^ examines what trade-offs exist concerning economic growth and climate change issues. This is the context through which LCD pathways for the West African electricity system is examined. It identifies four high leverage points that could serve to achieve LCD in the WAPP.

## Methodology

### Data collection

**Figure 4.**
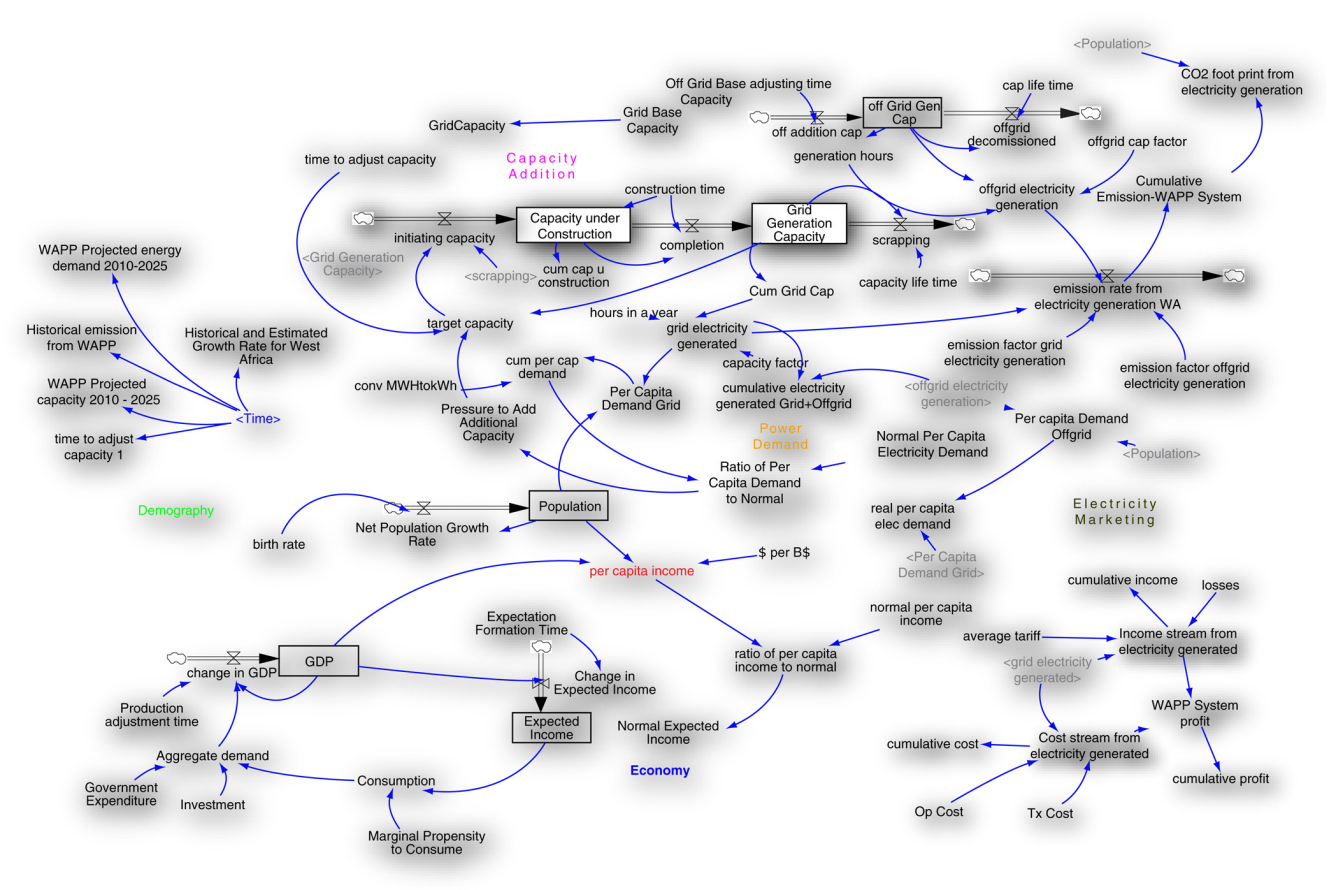
SD Electricity Planning-Low Carbon Development Model (adapted from Momodu *et al.* ^4^ Op cost: operational cost, Tx cost: Tax cost, MW* capacity, GWh**: power demand, GDP: gross domestic product. *MW = Megawatts, **GWh = gigawatt hours.

Vensim DSS version 6.2 was used to develop the model. WAPP member country level data elicited from WAPP and ECOWAS Regional Electricity Regulatory Authority (ERERA) serves as the set of basic data used to develop and run the main model ( Figure 4). Other data elicited from various journal articles and internet sources (Central Banks of ECOWAS Member State Countries; Bureau of Statistics; Electricity Regulatory Bodies) include population and its average growth rate, GDP, per capita income, average per capita electricity demand, electricity generated, average electricity tariff, generation technology type, amongst others. The generated SD model examines the (nonlinear) relationship regarding generation adequacy and GHG emission reduction in the WAPP. It evaluated the strain of providing adequate supply capacity as against emission reduction from the generation technologies in the West Africa electricity system. The complexities in the West African electricity system were arranged in the model to establish the basic interconnecting structure for the system analysis; this is to achieve global expectations of emission reduction and economic growth.

### Model development

SD Model for planning in the WAPP system is shown in Figure 4. The model development was guided with CLD and SFD as explained in earlier sections depicted in Figure 2 and Figure 3. Its structure was developed with the boundary set around electricity supply, generation and marketing, population and the GDP. This was achieved through WAPP electricity system operations review. The model focuses largely on interconnections in the WAPP power system regarding its operations and GHG emission. The power system examined emission from the basis of generation and average emission factor as well as based on generation technology types.

The first assumption to developing the model is that the different regulatory authorities in the WAPP system are responsible for reviewing proposals submitted for building of new power plants. Second assumption in order to ease the model development is that these authorities all have uniform *modus operandi*. The first step is that applications to construct new power plants by would-be investors accumulate to regulatory authorities for approval or rejection. The time needed to process proposals depends on both capacity of examining multiple projects and project complexities, such as proposed technologies (e.g. nuclear, hydro, CCGT) and the specific siting of the new power station. This process could take some time depending on the technology type being processed. The normal assumption is that when the permits are granted, would-be investor will construct the power plants due to inadequacy in the system currently. In order to achieve balancing feedback, time delay is assumed to be reduced to provide a higher stability margin to the system. Nevertheless, when new efficient plants in pipeline are finished and start to generate, prices may not be depressed for older plants as they are under long term contract and will not likely exit business. The effect of this is increase in the rate of aging in the available stocks, which has positive effect on the reserve margin. However, the growth of electricity demand is expected to impact on reserve margin negatively, causing a new wave of constructions due to both accumulated permits and a stream of new proposals. For accumulated permits, starts are immediate; stream of new proposals would have to face delay in the time needed to obtain permits. This implies that though decision of investing in new plants may be simultaneous, it will however have a different time-phase for the market place.

The stock-and-flow structure must be further expanded in parallel stock chains in order to consider the different characteristics of the several available technologies. Since the stocks represent different stages of the power plant operations such as the installed capacity, the capacity under construction, etc., this have to be disaggregated to account for different lifetimes, construction lead-times, permitting delays, etc. In addition to this disaggregation, for the same generating technology, installed capacity has to be distinguished by age to keep track of thermal efficiencies, and therefore, the spread in marginal cost of production.

**Figure 5.**
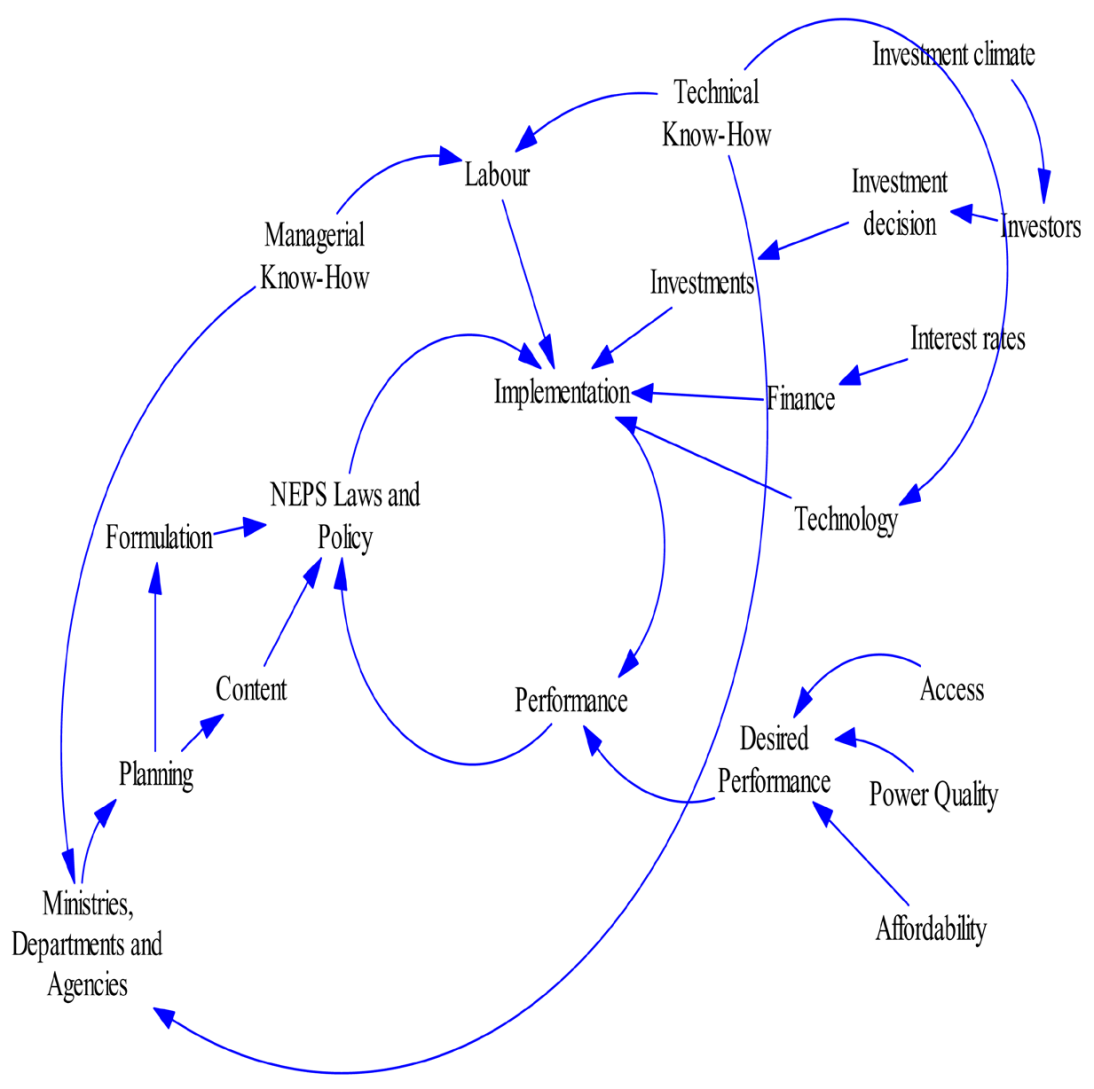
Causal-Loop Diagram of the NEPS adopted for WAPP System ^24^.

With the foregoing, the first step to developing the SD model is to assemble and analyze an array of data pertaining to the electric power sector in the WAPP (see 23). This step helped to understand the interconnections in the system that affects its performance. From these interconnections, is developed the causal-loop diagram ( Figure 5). This simplifies the development of stock and flow diagram that make up the model. With the stock and flow diagram, the set of equations driven the model to run were derived.

Models developed in Vensim are capable of using time frame of seconds to many years, depending on the kind of system being evaluated in the model. The time frame used for developing this model to evaluate the long-term performance of the WAPP system is 50 years, 2015 to 2064. This can be changed within the model setting. Evaluating the long-term performance of WAPP system requires developing a model that takes the dynamics of the structure and behaviour of the system into consideration. Having realized this need from the onset, SD technique was chosen using Vensim 6.2 software platform, which is one of the recognized software platforms with capability for developing such model ( http://www.vensim.com/sdmail/sdsoft.html; Wikipedia website). To develop this model, the main ideas made use of Ford study ^25, 26^ as well that in Oyebisi and Momodu ^23^. The model assumes that the WAPP will act as one market, where price of electricity would be driven be driven by demand and supply, that is, levelised energy cost as well as future retail tariff. Demand of electricity is based on WAPP forecasting data.

Figure 4 shows the causal loop diagram showing the interconnections in the Nigerian electricity system ^24^. This was adopted for the WAPP system. Following from knowing this structure and behaviour characteristics, the first step to developing the model was to make use of historical data available for performance of the system. These include its installed capacity (taking different technology in the generation park into consideration), availability factor, capacity factor, peak load, energy generated, transmission capacity, losses (transmission, distribution, non-technical and non-billed energy sent out), and energy sold. This formed the baseline information data to develop the model. Other parameters that were developed for the baseline include gross domestic product and population of the country. From the baseline information other variables which contribute to the structure and behaviour of the system were added. These include capacity addition, future capacity needed, and regulated tariff. Having developed the model based on the baseline information, other parameters needed were then calculated. This ensures both validation and robustness of the model to explain the behaviour of the WAPP system.

There are quite a number of variables for estimating future population depending on the finer details needed. However, the most striking variables are those of fertility and mortality rates ^27^. In this study, a simple approach of birth and death was used to develop a future population trend for Nigeria. Future birth per year of the existing population is a factor driven by birth rate, while death is regulated by average life expectancy. Mathematically, this is represented in the model as:

$$Population=\int_{t=1}^{t=36}\left(birth-death\right)$$

where

$$birth_{t+1}=birthrate_{t+1}\times Population_{t},\text{and}$$

$$death=average\;life\;expectancy_{t\;+1}\;\times Population$$

The dynamics of the model is then described by a set of non-linear differential equations that account for existing system feedbacks, delays, stock-and-flow structures and nonlinearities. What makes the model to stand out is that its various sectors and spheres are connected together to as a complex link of feedback loops. This allows the electric system to be analyzed and weighted as a driving or limiting factor for LCD agenda in any country.

A major principle in the developed SD model is the leverage points. “ *Leverage points are places within a complex system (a corporation, an economy, a living body, a city, an ecosystem) where a small shift in one thing can produce big changes in everything*” (see Donella Meadows Project page on leverage points). For SD model, the leverage points are points of importance in a system that modelers not only believe in but would be glad to know where they exist and how to locate them.

The “state of the system” — electricity, a nonmaterial commodity - in whatever standing stock is of importance. The stock is increased by inflow - electricity generation, investment and financial flow; while the outflow decreases it - transmission, distribution, losses and thefts. So, the bedrock of this system consists of physical stocks and flows, obeying the laws of conservation and accumulation. WAPP power system has inadequacy; simply interpreted to mean inflow rate is lower than the outflow rate. In other words, the non-storable commodity is always in shortfall.

Systems respond at a slower rate to desired growth rate as is typical for flows to accumulate. Electricity system in the WAPP exhibits the same characteristics; it will take time to correct the anomalies, being sluggish in responding to desired changes. This sluggishness is referred to as delay in the system. To achieve a quick turnaround in the system, it is critical to identify the 'leverage points' along the line of the operations of the WAPP electricity system. This takes into consideration the superimposed LCD strategy in the model as corrective measures to achieve change in the system.

At least two negative feedback loops, or correcting loops exist in a system ^13– 15, 28^; the first is what controls the inflow, and the second, controls the outflow. Either or both can be used to bring the system to a desired level. Usually, the goal and the feedback connections are not visible in the system except when viewed from long-term perspective to figure out what are the leverage points in it. In this study a new paradigm - LCD – was superimposed into the planning of an already complex WAPP system. This is with the desire for a future responsive plan amenable for delivering globally cost competitive electricity with reduced emission.

### Brief description of model workings

There are three interconnected sectors in the model. The sectors are electricity (split into capacity addition (MW) and power demand (GWh)), demography (principally the population) (persons), economy as depicted by the gross domestic product (GDP) (Billion US$), make up the three modules in the model. There are two sub-modules in the model as offshoot from the power demand segment under the electricity module. These are: emission from electricity consumption (tCO _2_) and electricity marketing (US$). Analysis of results from the model was limited to only the electricity module as data needed from other two modules narrowed the integrity of their results.

Each of the modules has at least one Level variable with integral equation. Most level variable equations in Vensim ^®^ software take the form of:

**Level Variable (Name) = ∫** ( *Inflow* ( *t*) – *Outflow* ( *t*), *initial* _ *value* ( *0*)

Level variables represent stocks in system dynamic models ^14, 15^. This means that all the Level variables in the model, namely, Population, GDP, Capacity under Construction, and Grid Generation Capacity are stock operating the Principle of Accumulation ^3^, and take on the form of Equation (1) to run in the model. Now, the critical aspect of Level variable is that it allows for the introduction of the concept of delay, a dynamic function, into the system being modeled. Being stocks, these variables have four important characteristics of having memory, changing the time shape of flows, decouple flows and create delays. The concept of delay is expatiated upon elsewhere ^22^. One of the principal equations is that of Grid Generation Capacity as represented thus:

**Grid Generation Capacity [WAPP Member Countries**]= ∫ (completion[WAPP Member Countries]-scrapping [WAPP Member Countries], Grid Base Capacity[WAPP Member Countries]), Units: MW.

What is placed in the [X] shows the subscripts to the equation. This allows one variable and equation to represent a number of different distinct concepts.

The grid segment has capacity under construction to reflect how capacity is increased over the years. The grid capacity segment also has scrapping, which is driven principally by capacity life time (Years). The critical aspect of the capacity under construction is the assessment of initiating capacity, which in turn is driven by target capacity (MW) and time to adjust (Years). The target capacity is driven by per capita power generation in the system. The per capita power generation (kWh/Person) in the system is assumed to be driven principally by population (this is derived from the demography segment of the model). (In a fully liberalized market, this is expected to be determined by investor behavior - e.g. see 22).

It is important to state that the modules in the model are subscripted. Subscript in the modules allowed a variable (e.g. grid capacity, birth rate, GDP, etc.) to represent more than just a data for the variable. In this model, the subscript dealt with WAPP member country level information as well as aggregation of generation technology types in the WAPP.

### Scenario development and sensitivity analysis

Parameters within the model forms the basis for developing the scenarios for analysis. For the description in this chapter, two scenarios on the WAPP electricity system, Base Case and LCD Options, are examined. The Base Case scenario represents continuing a "business-as-usual" approach that draws on technologies in the electricity system as they currently are. No consideration is given for efficiency and how these technologies fare in terms of contribution to global warming through emission of GHG into the atmosphere. In the LCD Options, higher efficiency technologies emitting low carbon are drawn upon to replace generation technologies with high emitting factors. The LCD Option is examined based on changes in two parameters, namely, capacity and emission factors, against two different values of per capita electricity generation levels respectively. Other parameters are kept constant as in Base Case Scenario. To improve on the WAPP system, the LCD Option 1 was assumed to have emission factor improved by 10%, meaning EF is reduced by a factor of 0.1 from that of the Base Scenario, for each of the plants, while for LCD Option 2, it is reduced by a factor of 0.3.

The model is made up of seven subscripted ^4^ parameters, with the Base Case values listed in Table 1 being country level data and Table 2 being aggregated technology type in the WAPP. The high leverage points for policy intervention were identified from Table 1 and Table 2. High leverage points are places within a complex system (a corporation, an economy, a living body, a city, an ecosystem) where a small shift in one thing can produce big changes in everything else ^29^. Further testing - sensitivity analysis - could be conducted on the model. The high leverage points are parameters in the model as constants to conduct the sensitivity analysis. Sensitivity testing is the process of changing assumptions about the value of constants in the model and examining the resulting output (see 2010 System Dynamics conference site).

**Table 1a.** Ranges of future generation capacity (MW *), electricity generation (MWh) and CO _2_ emissions (tCO _2_).

Description	Scenario	2015	2030	2064	
Cumulative generation capacity, MW	Base	9912	8974	13718	Result for the two LCD Option are the same despite differences in the CF and EF used
	LCD Option: 1	9912	12108	31049	
	LCD Option: 2	9912	12108	31049	
Cumulative electricity generated, Billion MWh	Base	29368	26535	40366	Result for the two LCD Option are the same despite differences in the CF and EF used
	LCD Option: 1	44051	53797	137838	
	LCD Option: 2	44051	53797	137838	
Cumulative CO2 Emission, Million tCO2	Base	15.3	13.8	20.9	The emissions from each of the scenario were different due to change in CF and EF respectively
	LCD Option: 1	18.3	22.5	57.1	
	LCD Option: 2	16.1	19.6	50	

*MW = Megawatts; MWh = Megawatt hour; CO _2_ – carbon dioxide; tCO _2_ = tons of carbon dioxide; LCD = low carbon development; CF = capacity factor; EF = emission factor;

**Table 1b.** Some assumptions made to estimate ranges of future generation capacity, electricity generation and CO _2_ emissions.

Assumptions			
Average per capita electricity consumption for WAPP countries, MWh/cap	Base	115	Reference year: 2015
	LCD Option: 1	363	
	LCD Option: 2	611	
Average capacity factor for countries, ratio	Base	0.54	Reference year: 2015
	LCD Option: 1	0.75	
	LCD Option: 2	0.81	
Average emission factor for WAPP countries, tCO2/MWh	Base	0.6526	
	LCD Option: 1	0.5221	
	LCD Option: 2	0.4568	
Expectation formation, years	Base	7	
	LCD Option: 1	7.5	
	LCD Option: 2	7.5	
Time to adjust capacity, years	Base	21	
	LCD Option: 1	21	
	LCD Option: 2	21	

WAPP: West African Power Pool; tCO2 = tons of carbon dioxide; MWh = Megawatt hour; LCD = low carbon development

**Table 2.** Emission projection from generated electricity.

Country	2015	2025	2035	2045	2055	2064
Benin_Base	226,100	185,959	211,318	240,135	272,881	306,154
Benin_LCD ^6^	271,320	285,769	387,195	524,617	710,814	934,283
Burkina_Base	280,384	219,006	239,190	261,235	285,311	308,871
Burkina_LCD	336,461	338,621	441,564	575,803	750,851	953,468
CDV_Base	1,124,299	871,223	890,825	910,868	931,362	950,201
CDV_LCD	1,349,159	1,323,769	1,614,082	1,968,063	2,399,674	2,868,496
Gambia_Base	94,182	66,948	61,158	55,869	51,037	47,047
Gambia_LCD	113,018	101,326	110,511	120,528	131,453	142,129
Ghana_Base	2,134,793	1,755,796	1,995,228	2,267,311	2,576,496	2,890,654
Ghana_LCD	2,561,752	2,698,184	3,655,822	4,953,344	6,711,383	8,821,332
Guinea_Base	273,443	213,584	233,269	254,768	278,248	301,225
Guinea_LCD	328,132	330,238	430,633	561,549	732,265	929,866
GuBis_Base	56,727	43,958	44,947	45,958	46,992	47,943
GuBis_LCD	68,073	66,792	81,440	99,300	121,077	144,732
Liberia_Base	38,472	30,050	32,820	35,844	39,148	42,381
Liberia_LCD	46,166	46,463	60,588	79,007	103,025	130,827
Mali_Base	149,490	110,531	104,188	98,210	92,574	87,779
Mali_LCD	179,388	166,577	187,260	210,512	236,650	262,937
Niger_Base	318,737	236,206	233,207	230,246	227,322	224,723
Niger_LCD	382,484	360,709	425,192	501,203	590,801	685,057

GuBis = Guinea Bissau; CDV = Cote d’Ivoire, LCD = low carbon development

Multivariate sensitivity simulation (MVSS) or Monte Carlo simulation is a natural choice where multiple parameters are identified in the model as high leverage points. Four high leverage points identified in the West African electricity system are capacity factor (CF), emission factor (EF) (country average and technology type), time to adjust capacity and expectation formation. In running of the model, two parameters stood out on their effect on generation capacity addition and GDP.

More description of the parameter “time to adjust capacity” can be found in Momodu ^2^.

## Result and analysis of model output based on different scenarios

Simulation result from running the model is presented in this section. The simulation was done as Base Case and LCD Options (1 and 2) respectively. Temporal variability used for the model was 50 years, starting with 2015 and terminating at 2064. After establishing the model structure and unit checks made, it was further validated by comparing the simulated output with empirical values gotten for WAPP in an independent study (see Nigerian government document on economic recovery and growth).

Detail description of expectation formation periods and time to adjust capacity are given in 2. In reality, these times (expectation formation and time to adjust) are missing in operating most of the power systems in the region. For example, out of the 24 documented power plants in Nigeria, only 14 were built as recent as 30 years or less ago. These power plants are only 48% of the total generation capacity, with no immediate plans of their replacement.

Table 1a shows the ranges of future generation capacity in MW with projected electricity to be generated from this capacity as well as the emissions measured in billion MWh and tonnes of CO _2 _respectively. Table 1b gives the assumptions made for each of the scenario options using 2015 as the reference year.

From the model run, weighted average emission of GHG was 27.1 million tCO _2_ equivalent for the Base Scenario in the 50-year period. For the two LCD Options, the average weighted average annual emission is between 31.5 and 15.8 million tCO _2_ equivalent respectively. The cumulative emission for the scenarios are 1.4, 2.2 and 0.8 billion tCO _2_ respectively. Adopting the strategy of improved capacity and emission factors of these aged plants achieved significant reduction in emission levels as seen in the LCD option 2.

### Low Carbon Development Strategy in WAPP

To achieve development, energy consumption would need to be increased. This means expansion of grid capacity in generation, transmission and distribution. So, at first glance as shown in Table 2, what is gleaned from the SDM is that development policies will be running counter to reducing GHG emission. Thus, to counter such direction it becomes necessary to apply a strategy that procures low carbon. This strategy will handle tension between achieving development and reducing GHG emissions from infrastructural provision. LCDS then becomes a means to achieve balancing of factors for policies that affect development and those that meant for climate change control. LCDS is counterintuitive, because it recognizes the existence of negative feedback loops. This is demonstrated in the result shown in Table 2. The Base Scenario guided how mitigation targets were set.

To examine low hanging options available for achieving LCDS in the WAPP electricity system, the two other alternatives were run for the LCD Option, namely, LCD Option 1 and 2 respectively. The first alternative considered increased efficiency in the generation capacity by a factor of 50%, through improved average capacity factor across the countries.

The second alternative, considered reduced emission through reducing average emission factor across WAPP member by 30%. This is in addition to the strategy adopted in LCD option 1. In the first alternative, the system gained increased energy generation, though with corresponding increase in emission as shown in Table 3. Still on Table 3, for LCD Option 2, when the average emission released from these plants were improved upon through across board 30% reduction in the emission factors, a reduced emission was achieved compared to merely improving the capacity factor. The energy generated in both alternatives, however, were similar. These two low-hanging alternatives analyzed were first identified as high leverage points from conducting the base run simulation.

**Table 3.** Results of running the EP-LCDv1 * Model at different CF and EF respectively.

				Difference
	Scenario	2015	2064	50
		Million tCO _2_		
	Base (A)	15.28	20.9	5.62
LCD Options	LCD-CF0.5>Base_EFBase (B)	22.93	71.37	48.44
	LCD-CF0.5>Base_EF0.3<Base (C)	18.34	57.1	38.76

*EP-LCDv1 = Electricity Planning-Low Carbon Development Model version 1, LCD = low carbon development; CF = capacity factor; EF = emission factor; tCO _2_ = tons of carbon dioxide

The strategic intervention examined is for improved capacity and emission factors respectively. All other identified parameters are kept at their Base run values. For the electricity sector, this intervention is seen as low hanging option to address the trade-offs between economic development and emission reduction as options for targeting low carbon economy in West Africa. The possible barriers to achieving this intervention include but not limited to the following:

(1) not being ready to promote the technical knowhow needed to achieve desired improvement in the capacity and emission factor levels amongst the existing power generation technologies across the nations in WAPP;
(2) not willing to incentivize the sector to attract potential investors and innovators with adequate reward. Innovation means practices amongst the stakeholders in the WAPP system that generate electricity, to have desirable features in line with global emission reduction objectives, as in the Paris Agreement.

To estimate the cost impact, only existing technologies were examined without taking into consideration environmental factor improvement such as carbon capture storage alongside the generation of new technology. Further, the estimate of cost impact was based on an across board average having combined the overnight cost ^28, 29^ for all existing generation technologies in West Africa, namely, oil, natural gas, coal and hydro.

The cost impact was calculated based on construction of new power plants. Factors determining new power plants cost are: environmental regulations, construction costs, financing costs and fuel expense. Other factors that drive power plants cost are government incentives, air emissions control on coal and natural gas.

**Figure 6.**
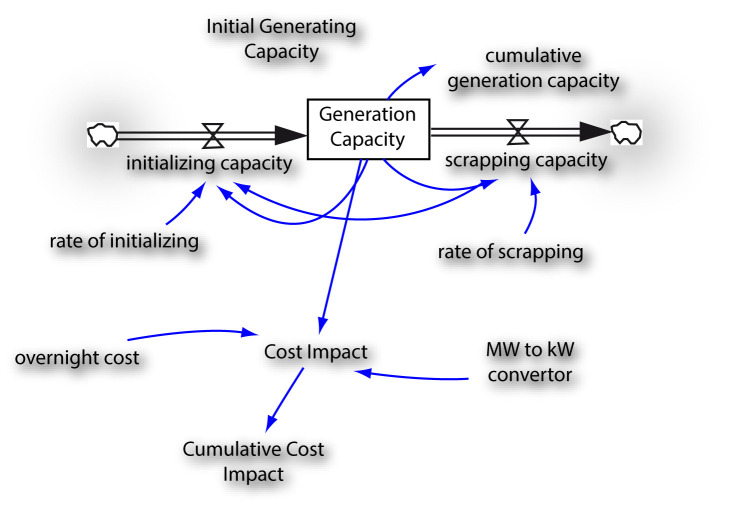
Cost impact assessment model for West African Power Pool (WAPP) energy-climate change trade-offs. MW = Megawatts, kW = kilowatts.

Figure 6 is the mini model developed to assess the cost impact of these trade-offs. It was assumed that compounded annual initializing (growth) rate and compounded annual scraping rate in the system will be 6% and 1.5% respectively. Cost values were taken from 29, 30. Table 4 shows the cost impact for 2064. Total cumulative cost impact is approximately US$1.54 trillion from 2018 through to 2064. The cost impact was limited to capital, financing and fuel costs ^27, 28^ to estimate what is needed to achieve trade-offs in the WAPP system. This means that the total cost will be significantly higher than what is estimated here.

**Table 4.** Cost impact of generation capacity in the WAPP system in 2064.

Technology/ Fuel Type	Overnight Cost, $/kW (2012$)	Capacity in 2064, MW	Cost implication, US$, Billion
Oil-Fired	1200	4027	4.84
Natural Gas	1023	13,970	14.30
Coal	3246	91.4	0.30
Hydro	2936	10,740	31.54
**Total**			50.98

Sources: ^31– 33^ kW: kilowatt, MW: megawatt

It is pertinent to point out that the cost estimates are based on OECD standard. The cost of this same technologies from other regions may be more competitive. Now, the existing mix of generation technologies in the WAPP system consists of oil-fired, natural gas, coal and hydro plants. The generators are not new and they have aged, and over the years, have been affected by changes in technology and economics. Indeed, most of the plants in the WAPP system have units that were built decades ago as base-load stations. Though still being operated as base-load ^5, 31^ (see Emission Factors site), they are best to be described as cycling or peaking plants. They have high fuel prices and poor efficiency, making them economically marginal. This implies that the regional organization will need to get the government of WAPP member nations involved to incentivize the strategic intervention process to for LCDS.

The government in this region must decide to deliberately influence the factors affecting cost of electricity to determine the kind of improvement that could be achieved in existing plants and/or power plants that would be built in the future. The first step would be to encourage the policy of energy efficiency through technological improvement with increased capacity factor and decreased energy intensity of economic activities that will also mean reduced emission factor. To encourage energy efficiency and decrease energy intensity of economic activities will both require increased spending on research and development on the part of government. This is currently non-existent in the West African region. The second strategy should be aimed at incentivizing construction of power plants to especially benefit base-load plants such as those that will encourage emission reduction, which are costly to build. The third approach which will be short term, say in a five-year period, that will allow low carbon fuels (principally, natural gas) cost to benefit intermediate load plants as a transition process; though inexpensive to build they have high variable cost due to fuel expense. This is to encourage a quick ramp up of generation capacity to increase economic dispatch in the grid system. These strategies should however be reviewed periodically in line with determination to meet Paris Agreement on NDC for these nations.

## Summary and conclusion

The primary goal of this paper was to demonstrate the use of SDM as an inexpensive means to examine low carbon pathway. This was achieved with the recommendation of four strategies to encourage the implementation of energy efficiency policies as regards emission reductions. These are: a) enforcement of improved efficient electricity generation through increased energy efficiency that should result in increased capacity factor. This could be achieved through incentivizing retrofitting process; b) decreased energy intensity of the economy that should result in reduced emission factor amongst existing plants. This strategy involves rehabilitation of the existing installations to elongate the lifespan of aged power plants in improved forms; c) attract new investment through low tax or tax exemption to reduce the cost of construction that will benefit base-load plants. This is to attract investment in new generations that encourage low carbon economy in the WAPP system; and d) subsidized cost of low-carbon fuels in the short run to benefit intermediate load plants and allow for the ramping up of low-/no-carbon fuel generation capacity. This is considering that building new power generation facilities and transmission lines need much more significant resources than improving on their reintegration, namely, returning the facility back to its former operational condition. These approaches are recommended considering the region’s specific economic and political conditions; funds are tremendously difficult to raise ^26^. Implementing these recommendations will allow the electric power industry in West Africa to contribute to achieving sustainable development path.

It is pertinent to point out the use of System Dynamics was basically intended to understand the behaviour of the system to be able to conduct any intervening action on it. This study had examined factors that brought out some values in the complex social system of climate change and economic development connected by energy use affect humans. On the one hand, energy is needed to drive economic development, while on the other hand, there energy use is a major driving force behind climate change. Policy makers then cannot just use values to step in and set about fixing the problem. This may portend greater risk in the complex system. Meddling in one part of the complex system may result in almost certain risk of setting off disastrous events that had not been counted on in other, remote parts of the system. Therefore, fixing the push-pull factor of climate change-economic development interaction where energy use is needed, demand that policy makers first understand the whole system thoroughly ^32, 33^.

## Data availability

The data underlying this study is available from Open Science Framework. Dataset 1: System dynamic modelling of electricity planning and climate change in West Africa. http://doi.org/10.17605/OSF.IO/2AM9T ^34^

This dataset is available under a CC0 1.0 Universal licence

## Footnotes

^1^ This simply refers to the path that has had predominantly fossil-fuel based energy technologies.

^2^ Autarkical refers to something that is free from external control and constraint, or independent; while integration refers to something dependent or not constrained.

^3^ Accumulate: growing or increasing over time http://www.eia.gov/todayinenergy/detail.cfm?id=22832#

^4^ Subscript is any variable representing more than one distinct concept.

^5^ **Base-load** plants such as nuclear, coal, and geothermal base-load units, are expensive to build but have low fuel costs and therefore low variable costs. Other than for planned and forced maintenance, these generators will run throughout the year. **Intermediate load** plants, such as combined cycle units, are very efficient but use expensive natural gas as a fuel. These cycling plants will ramp up and down during the day and will be turned on and off dozens of times a year. **Peaking load** plants, use combustion turbines and are relatively inefficient and burn expensive natural gas. They run only as needed to meet the highest loads. (See Emission Factors site).

## References

[1] NERC: Multi-Year Tariff Order for the Determination of the Cost of Electricity Generation for the Period 1 June 2012 to 31 May Nigerian Electricity Regulatory Commission 1 ^st^ JUNE 2012.2017;1–37. Reference Source

[2] Momodu AS Addo A Akinbami JFK : Low-carbon development strategy for the West African electricity system: preliminary assessment using System dynamics approach. *Energy Sustain Soc.* 2017;7(11):1–23. 10.1186/s13705-017-0113-4

[3] Moumouni Y Ahmad S Baker RJ : A system dynamics model for energy planning in Niger. *Int J Energy Power Eng.* 2014;3(6):308–322. 10.11648/j.ijepe.20140306.14

[4] Ford A : System dynamics and the electric power industry. *Syst Dyn Rev.* 1997;13(1):57–85. 10.1002/(SICI)1099-1727(199721)13:1<57::AID-SDR117>3.0.CO;2-B

[5] Sterman JD : Business Dynamics: System Thinking and Modeling for a Complex World.Irwin McGraw-Hill, Boston, MA, USA - ISBN 0-07-231135-5.2000. Reference Source

[6] Raupach MR Marland G Ciais P : Global and regional drivers of accelerating CO _2_ emissions. *Proc Natl Acad Sci U S A.* 2007;104(24):10288–10293. 10.1073/pnas.0700609104 17519334 PMC1876160

[7] Bert MO Davidson P Dave BR : Climate Change Mitigation. *Contribution of Working Group III to the fourth assessment report of the Intergovernmental Panel on Climate Change*. 2007. Reference Source

[8] Kojima M Robert B : Changes in CO _2 _Emissions from Energy use: A Multicountry Decomposition Analysis.The World Bank, Washington DC, USA.2009. Reference Source

[9] Mundaca L Markandya A Nørgaard J : Walking away from a low-carbon economy? Recent and historical trends using a regional decomposition analysis. *Energ Policy.* 2013;61:1471–80. 10.1016/j.enpol.2013.04.083

[10] Arto I Dietzenbacher E : Drivers of the growth in global greenhouse gas emissions. *Environ Sci Technol.* 2014;48(10):5388–94. 10.1021/es5005347 24754816

[11] McLUCAS AC : Incorporating soft variables into system dynamics models: a suggested method and basis for ongoing research.In *Proceedings of the 21st international conference of the System Dynamics Society*.2003;20–24. Reference Source

[12] Feng YY Chen SQ Zhang LX : System dynamics modeling for urban energy consumption and CO _2_ emissions: A case study of Beijing, China. *Ecol Modell.* 2013;252:44–52. 10.1016/j.ecolmodel.2012.09.008

[13] Sterman JD : System dynamics modeling: tools for learning in a complex world. *Calif Manage Rev.* 2001;43(4):8–25. 10.2307/41166098

[14] Þorsteinsson RV : A brief introduction to System Dynamics [Online]. [Accessed: 8 November 2017]. Reference Source

[15] Vlachos D Patroklos G Eleftherios I : A system dynamics model for dynamic capacity planning of remanufacturing in closed-loop supply chains. *Comput Oper Res.* 2007;34(2):367–394. 10.1016/j.cor.2005.03.005

[16] Kivuti-Bitok L Pokhariyal GP Momodu AS : Cervical Cancer Management in Developing Countries: A System Dynamics Modelling Approach.In: dall’Acqua (Ed) *Froecasting and managing risk in health and safety sectors*. IGI Global Publisher (in press).

[17] Olsina F : Long-Term Dynamics of Liberalized Electricity Markets. *Thesis submitted to Department of Postgraduate Studies, Faculty of Engineering*, National University of San Juan, San Juan, Argentina, accessed on September 8, 2008,2005. Reference Source

[18] Gupta S Tirpak DA Burger N : Policies, instruments and co-operative arrangements.In: B. Metz, OR. Davidson, PR. Bosch, R. Dave, LA. Meyer (eds), *Climate Change 2007: Mitigation. Contribution of Working Group III to the IPCC Fourth Assessment Report*, Cambridge, UK Cambridge University Press,2007;745–807. Reference Source

[19] Pachauri RK Allen MR Barros VR : Climate change 2014: synthesis report. Contribution of Working Groups I, II and III to the fifth assessment report of the Intergovernmental Panel on Climate Change. IPCC,2014. Reference Source

[20] Gnansounou E Bayem H Bednyagin D : Strategies for regional integration of electricity supply in West Africa. *Energ Policy.* 2007;35(8):4142–4153. 10.1016/j.enpol.2007.02.023

[21] Gebremicael M Yuan H Tomsovic K : Use of system dynamics for studying a restructured West African power pool. *2009 IEEE Power & Energy Society General Meeting.* Calgary, AB,2009;1–4. 10.1109/PES.2009.5275368

[22] Aissatou N Rabani A Moussa G : Global Warming and Heat Waves in West-Africa: Impacts on Electricity Consumption in Dakar (Senegal) and Niamey (Niger). *International Journal of Energy and Environmental Science.* 2017;2(1):16–26. Reference Source

[23] Oyebisi TO Momodu AS : Planning for high technology facility in Nigeria: a perspective. *Int J Technology Policy and Management.* 2012;12(1):48–57. 10.1504/IJTPM.2012.044968

[24] Momodu AS : Evaluation of Long-term Performance of the Electric Power System in Nigeria.Ph.D. Thesis, Obafemi Awolowo University, Ile-Ife.2012.

[25] Ford A : Modeling the Environment: An Introduction to System Dynamics Modeling of Environmental Systems.Island Press, Washington DC,1999;1(1):401. 10.1108/ijshe.2000.24901aae.002

[26] Ford A : System dynamics and the electric power industry. *Syst Dyn Rev.* Jay Wright Forrester Prize Lecture.1997;13(1):57–85. 10.1002/(SICI)1099-1727(199721)13:1<57::AID-SDR117>3.0.CO;2-B

[27] Ohio Research Office: A State Affiliate of US Census Bureau.Population Projection Methodologies.2013. Reference Source

[28] Kilanc PG Or I : A decision support tool for the analysis of pricing, investment and regulatory processes in a decentralized electricity market. *Energy Policy.* 2008;36(8):3036–3044. 10.1016/j.enpol.2008.03.034

[29] U.S. Energy Information Administration: Updated Capital Cost Estimates for Utility Scale Electricity Generating Plants.U.S. Energy Information Administration;2013;524. Reference Source

[30] Kaplan S : Power Plants: Characteristics and Costs.DIANE Publishing, Darby, Pa USA;2011. Reference Source

[31] Brander M Sood A Wylie C : Technical paper | Electricity-specific emission factors for grid electricity.Ecometrica, Emissionfactors. com.2011. Reference Source

[32] Sterman JD : Business dynamics: systems thinking and modeling for a complex world.2000. Reference Source

[33] Thomas L : The Lives of a Cell: Notes of a Biology Watcher.New York: Viking Press.1974. Reference Source

[34] Momodu AS : System dynamic modelling of electricity planning and climate change in West.2018. 10.17605/OSF.IO/2AM9T PMC9648363 36420048

