# Supplementary material for: System dynamic modelling of electricity planning and climate change in West Africa
Source: AAS Open Res. 2018 Oct 4;1:15. Originally published 2018 May 2. [Version 2] doi: 10.12688/aasopenres.12852.2 (PMC9648363; doi:10.12688/aasopenres.12852.2)
Supplement: Peer review history

# Peer review: Reviewer response for version 2

**DOI:** 10.21956/aasopenres.13947.r26623

**Published:** 2018-10-26

**Authors:** David Rees

**Affiliations:** 1 Synergia Ltd., Auckland , New Zealand

I note the changes made, which have made the article more concise and readable.

 I would however get someone to give it a close read as there are still a number of grammatical errors in the report.  Also, I would suggest rethinking the description of future 4 which is more of an influence diagram than a CLD, as there is only one feedback loop in it.

Is the work clearly and accurately presented and does it cite the current literature?

Partly

If applicable, is the statistical analysis and its interpretation appropriate?

Not applicable

Are all the source data underlying the results available to ensure full reproducibility?

Partly

Is the study design appropriate and is the work technically sound?

Partly

Are the conclusions drawn adequately supported by the results?

Partly

Are sufficient details of methods and analysis provided to allow replication by others?

No

Reviewer Expertise:

Application of System Dynamics in health, social services and energy.

I confirm that I have read this submission and believe that I have an appropriate level of expertise to confirm that it is of an acceptable scientific standard.

# Peer review: Reviewer response for version 1

**DOI:** 10.21956/aasopenres.13918.r26457

**Published:** 2018-06-25

**Authors:** David Rees

**Affiliations:** 1 Synergia Ltd., Auckland , New Zealand

This paper focuses on an important topic, well suited to the System Dynamics method. However, the paper does need substantial revision.

 The paper makes a number of strong assertions, that need to be supported with references or further explanation.  For example p3, para 1, L1, "Energy, particularly in electrical form, is one the most important value-adding commodities to sustainable development." This example also highlights the need for a close reading to fix grammatical errors.

 The paper also makes a number of assertions that are simply wrong.  For example, page 4, para 1, L7, "Stocks are accumulations of anything that can be counted." The authors seem to confuse measurement (counting) with quantification. Accumulations such as 'morale' may not be able to be measured, but they can certainly be quantified and the ability to include such 'soft' variables in a modelling effort is one of the great attributes of SD modelling.

 My biggest concern however is that the methods section does not really describe the method, and does not seem to follow a standard process. There are well documented approaches to developing SD models which at a minimum would include depiction of a 'reference behaviour', discussion of boundary issues, description of a conceptual model (often in the form of a CLD) and then discussion of the simulation model itself. They talk about CLDs but do not include any. There are also no reference behaviours. The result is that the method section does not really discuss method, beginning instead with a description of the model, not how it was developed.   Without these initial steps, discussion of the model becomes abstract and difficult to evaluate. At the very least the authors need to show a CLD, that describes the key feedback loops driving the system. that will be tested in the simulation. 

 My key recommendations are:

1. Re-write the methods section describing HOW the model was built, key assumptions, hypotheses etc.  Include reference behaviours and CLDs in that discussion.
2. Change your outputs from tables to runtime charts - as they better show changes over time, and make sure those outputs relate to the dynamic hypotheses you are making and describing in the CLDs.
3. Make sure all key assertions are supported with references and/or good argument.
4. Read through the paper closely to fix grammatical errors.

Is the work clearly and accurately presented and does it cite the current literature?

Partly

If applicable, is the statistical analysis and its interpretation appropriate?

Not applicable

Are all the source data underlying the results available to ensure full reproducibility?

Partly

Is the study design appropriate and is the work technically sound?

Partly

Are the conclusions drawn adequately supported by the results?

Partly

Are sufficient details of methods and analysis provided to allow replication by others?

No

Reviewer Expertise:

Application of System Dynamics in health, social services and energy.

I confirm that I have read this submission and believe that I have an appropriate level of expertise to confirm that it is of an acceptable scientific standard, however I have significant reservations, as outlined above.

# Response

**Published:** 2018-09-28

**Authors:** Abiodun Suleiman Momodu

**Affiliations:** Obafemi Awolowo University / Centre for Energy Research and Development, Nigeria

This paper focuses on an important topic, well suited to the System Dynamics method. However, the paper does need substantial revision.

 The paper makes a number of strong assertions, that need to be supported with references or further explanation.  For example p3, para 1, L1, "Energy, particularly in electrical form, is one the most important value-adding commodities to sustainable development." This example also highlights the need for a close reading to fix grammatical errors.

 Corrected.

 The paper also makes a number of assertions that are simply wrong.  For example, page 4, para 1, L7, "Stocks are accumulations of anything that can be counted." The authors seem to confuse measurement (counting) with quantification. Accumulations such as 'morale' may not be able to be measured, but they can certainly be quantified and the ability to include such 'soft' variables in a modelling effort is one of the great attributes of SD modelling.

 Corrected.

 My biggest concern however is that the methods section does not really describe the method, and does not seem to follow a standard process. There are well documented approaches to developing SD models which at a minimum would include depiction of a 'reference behaviour', discussion of boundary issues, description of a conceptual model (often in the form of a CLD) and then discussion of the simulation model itself. They talk about CLDs but do not include any. There are also no reference behaviours. The result is that the method section does not really discuss method, beginning instead with a description of the model, not how it was developed.   Without these initial steps, discussion of the model becomes abstract and difficult to evaluate. At the very least the authors need to show a CLD, that describes the key feedback loops driving the system. that will be tested in the simulation. 

 Corrected.

 My key recommendations are:

1. Re-write the methods section describing HOW the model was built, key assumptions, hypotheses etc.  Include reference behaviours and CLDs in that discussion. Done.
2. Change your outputs from tables to runtime charts - as they better show changes over time, and make sure those outputs relate to the dynamic hypotheses you are making and describing in the CLDs. Done.
3. Make sure all key assertions are supported with references and/or good argument. Done
4. Read through the paper closely to fix grammatical errors. Done

# Peer review: Reviewer response for version 1

**DOI:** 10.21956/aasopenres.13918.r26456

**Published:** 2018-06-11

**Authors:** Yacouba Moumouni

**Affiliations:** 1 University of Nevada, Las Vegas, Las Vegas, NV, USA; 2 Higher Colleges of Technology, Ras Al Khaimah, United Arab Emirates

As a reviewer, I appreciate the overall work. Couple of things that I would like to comment on.

 1)  Technically, the authors should review their definition of stocks because they forgot to include the intangible things, such as belief, thoughts, etc. Though they did mention countable things. Also, their use of commas and semicolons is at times questionable without any major prejudice on the meanings.

 2) To the question: Are sufficient details of methods and analysis provided to allow replication by others?

 This part is somewhat a little broad as System Dynamics encourages analyst to focus on specific problem rather than a whole region. It would have been much clearer had they focused on a single country.

Is the work clearly and accurately presented and does it cite the current literature?

Yes

If applicable, is the statistical analysis and its interpretation appropriate?

Not applicable

Are all the source data underlying the results available to ensure full reproducibility?

Yes

Is the study design appropriate and is the work technically sound?

Partly

Are the conclusions drawn adequately supported by the results?

Yes

Are sufficient details of methods and analysis provided to allow replication by others?

Partly

Reviewer Expertise:

Energy Storage Applications, Grid-tie Concentrated PV 7700 series, Thermoelectric Generators for Energy Delivery to Remote Residential Areas in Developing Regions, Wind power generation, and Engineering Education, Energy Supply and Demand Modeling Utilizing System Dynamics.

I confirm that I have read this submission and believe that I have an appropriate level of expertise to confirm that it is of an acceptable scientific standard.

# Peer review: Reviewer response for version 1

**DOI:** 10.21956/aasopenres.13918.r26381

**Published:** 2018-05-29

**Authors:** Dillip Kumar Das

**Affiliations:** 1 Department of Civil Engineering, Central University of Technology, Bloemfontein, South Africa

The intent of the paper is appreciable and the research theme is relevant. However, the paper lacks clarity and soundness. The authors must revisit their objective of the study as System Dynamics (SD) modelling is an established methodology/ modelling technique whose capabilities have already been demonstrated in different fields of study.

 The other flaws in the study is the way the methodology is presented. The methodology is not well articulated. The authors have mostly presented generic things. The methodology should focus on the current work.

 Further, there are no causal loop diagrams (CLD) or feedback relations provided to understand the conceptualisations of the model. The CLDs should form the backbone - dynamic hypotheses  based on which the model building should be done.

 It is also  to note that SD generates behaviours of the system not values. The authors have made the discussions based on the values and the behaviours were relegated.

 Overall the paper is confusing and not well articulated and needs major rework before it becomes fit for indexing. The authors should read a few papers on SD application that are available in the main stream of SD literature.

Is the work clearly and accurately presented and does it cite the current literature?

Partly

If applicable, is the statistical analysis and its interpretation appropriate?

Not applicable

Are all the source data underlying the results available to ensure full reproducibility?

No source data required

Is the study design appropriate and is the work technically sound?

Partly

Are the conclusions drawn adequately supported by the results?

Partly

Are sufficient details of methods and analysis provided to allow replication by others?

Partly

Reviewer Expertise:

NA

I confirm that I have read this submission and believe that I have an appropriate level of expertise to confirm that it is of an acceptable scientific standard, however I have significant reservations, as outlined above.

# Response

**Published:** 2018-05-29

**Authors:** Abiodun Suleiman Momodu

**Affiliations:** Obafemi Awolowo University / Centre for Energy Research and Development, Nigeria

Dear Reviewer,

Thank you for the incisive review on the work. We will work on the comments and revise as necessary.

Kind regards,

# Response

**Published:** 2018-09-28

**Authors:** Abiodun Suleiman Momodu

**Affiliations:** Obafemi Awolowo University / Centre for Energy Research and Development, Nigeria

The intent of the paper is appreciable and the research theme is relevant. However, the paper lacks clarity and soundness. The authors must revisit their objective of the study as System Dynamics (SD) modelling is an established methodology/ modelling technique whose capabilities have already been demonstrated in different fields of study.

The objective of the study was revised based on your comment.

The other flaws in the study is the way the methodology is presented. The methodology is not well articulated. The authors have mostly presented generic things. The methodology should focus on the current work.

The methodology has been cleaned up and made to reflect current work.

Further, there are no causal loop diagrams (CLD) or feedback relations provided to understand the conceptualisations of the model. The CLDs should form the backbone - dynamic hypotheses based on which the model building should be done.

Causal loop diagram has been added and explained.

It is also to note that SD generates behaviours of the system not values. The authors have made the discussions based on the values and the behaviours were relegated.

The behaviours have also been reflected in the discussion.

Overall the paper is confusing and not well articulated and needs major rework before it becomes fit for indexing. The authors should read a few papers on SD application that are available in the main stream of SD literature.

Suggestion well taken and followed in the review of the submitted work.
